# Peptide probes derived from pertuzumab by molecular dynamics modeling for HER2 positive tumor imaging

**DOI:** 10.1371/journal.pcbi.1005441

**Published:** 2017-04-13

**Authors:** Xiaoliang Yang, Zihua Wang, Zhichu Xiang, Dan Li, Zhiyuan Hu, Wei Cui, Lingling Geng, Qiaojun Fang

**Affiliations:** 1 CAS Key Laboratory for Biological Effects of Nanomaterials & Nanosafety, National Center for Nanoscience and Technology, Beijing, China; 2 CAS Center for Excellence in Nanoscience, National Center for Nanoscience and Technology, Beijing, China; 3 University of Chinese Academy of Sciences, Beijing, China; 4 School of Chemistry and Chemical Engineering, University of Chinese Academy of Sciences, Beijing, P.R. China; 5 Beijing Key Laboratory of Ambient Particles Health Effects and Prevention Techniques, Beijing, China; Max Planck Institute for Biophysical Chemistry, GERMANY

## Abstract

A high level of HER2 expression in breast cancer correlates with a higher tumor growth rate, high metastatic potential, and a poor long-term patient survival rate. Pertuzumab, a human monoclonal antibody, can reduce the effect of HER2 overexpression by preventing HER2 dimerization. In this study, a combination protocol of molecular dynamics modeling and MM/GBSA binding free energy calculations was applied to design peptides that interact with HER2 based on the HER2/pertuzumab crystal structure. Based on a β hairpin in pertuzumab from Glu46 to Lys65—which plays a key role in interacting with HER2—mutations were carried out *in silico* to improve the binding free energy of the hairpin that interacts with the Phe256-Lys314 of the HER2 protein. Combined the use of one-bead-one-compound library screening, among all the mutations, a peptide (**58F63Y**) with the lowest binding free energy was confirmed experimentally to have the highest affinity, and it may be used as a new probe in diagnosing and treating HER2-positive breast cancer.

## Author summary

Many therapeutic approaches, including the human monoclonal antibodies trastuzumab and pertuzumab, target the human epidermal growth factor receptor 2 (HER2) of any breast cancer that features HER2 overexpression. Compared to these antibodies, peptides have many advantages, including lower cost, easier synthesis, high affinity, and lower toxicity. Here, we first designed peptides that interact with HER2 protein based on the HER2/pertuzumab crystal structure (PDB entry: 1S78), using a combination protocol of molecular dynamics modeling, molecular mechanics/generalized Born solvent-accessible surface area (MM/GBSA) binding free energy calculations. Then, combined with the peptide library screening, six peptides were selected for further analysis and experimental validations. The results of *ex vivo* and *in vivo* experiments confirmed that one peptide (**58F63Y**) in particular has a strong affinity and high specificity to HER2-overexpressing tumors. This may due to more paired residues and lower binding free energy in peptide **58F63Y** and HER2 complex based on free energy decomposition analysis and distances calculation. While both *in silico* and *in vitro* screenings point to the same high-affinity peptide, the findings suggest that *in silico* screening based on calculated binding free energies is rather reliable. Additionally, based on the calculation of binding free energies among mutants, we can reduce the library capacity of one-bead-one-compound screening. In summary, we present a rather simple and rapid means of deriving a peptide with a clear binding site to its target protein.

## Introduction

Human epidermal growth factor receptor 2 (HER2) is a prominent target in breast cancer diagnosis and treatment, as approximately 20–30% of patients with breast cancer overexpressing the HER2 receptor [1,2], a 185-kD transmembrane glycoprotein with 1,255 amino acids [3]. The HER2 gene is a proto-oncogene that maps to chromosome 17q21. HER2 contains four domains (I, II, III, and IV) that comprise a ligand-binding extracellular portion, a single transmembrane helix, a tyrosine kinase domain closely related to the Janus kinases, and a C-terminal tail with a number of tyrosine phosphorylation sites that serve as a scaffold for adaptor molecules and enzymes in facilitating downstream signaling [4]. The heterodimerization of HER2 with any of the other three HER family receptors results in autophosphorylation of the terminal carboxyl segment and initiates a variety of signaling pathways that regulate cell growth, proliferation, and metastasis [5–7].

Currently, a number of therapeutic approaches have been developed to antagonize the effects of HER2 overexpression; these approaches include the humanized monoclonal antibodies trastuzumab and pertuzumab [8]. Trastuzumab demonstrates clinical benefits in the treatment of HER2-positive breast cancer, in both early and metastatic stages. One year of trastuzumab therapy is recommended for all patients with HER2-positive breast cancer who are also receiving chemotherapy [9]. However, as trastuzumab becomes a routine therapy, resistance can develop following an initial robust response; a lack of response to initiation has also been observed among patients [10,11]. The other antibody drug, pertuzumab, has received US Food and Drug Administration approval for the treatment of HER2-positive metastatic breast cancer. Trastuzumab and pertuzumab bind to different epitopes in the extracellular domain of HER2, and their mechanisms of action differ. Pertuzumab binds the pocket of domain II, inhibits HER2 dimerization with other receptors, and leads to slowed tumor growth. Trastuzumab, on the other hand, binds to subdomain IV [12], and works by inhibiting the PI3K/Akt, Mirk, and hKIS pathways and promoting proteolytic cleavage of the extracellular domain [13]. However, both drugs have been shown to stimulate the antibody-dependent cellular cytotoxicity mechanism [14].

It has been commonly recognized that, compared to antibody drugs, small peptides are cost-effective, have good tissue and membrane permeability, high target specificity, and low toxicity. Moreover, specific modifications to targeting peptides can be employed to provide diverse biosensing functions; this strategy has been leveraged to develop a method by which to detect metastatic tumor cells in primary tumors [15].

The science of molecular dynamics (MD) has been widely applied to chemical physics, materials science, and the modeling of biomolecules—such as interactions between ligands and receptors [16,17]—by simulating the physical movement of atoms and molecules based on a family of molecular mechanics force fields. The MM/GBSA method is often used to estimate the free energy of solute–solvent interactions. In this method, a generalized Born (GB) model is used to approximate the Poisson–Boltzmann equation, based on modeling a molecule as a set of spheres. The accessible surface area (SA) approximates the experimental value of the averaged behavior of many highly dynamic solvent molecules between the transfer free energy and the surface area of a solute molecule [18].

The one-bead-one-compound (OBOC) [19–21] library method can be used to systematically synthesize and screen the peptide library of a target protein. It is a simple means of rapidly identifying small molecules that bind with high affinity to receptor molecules. The strategy has been modified by many researchers to overcome the several limitations inherent in the original approach [22]. Our research group has previously advanced it to a lab-on-chip system that embraces the whole peptide screening process—from single bead trapping to the final sequencing of peptides—by using MALDI–TOF–MS [23,24].

In this study, we use a combination protocol comprising MD, MM/GBSA binding free energy calculation [25–27] to derive peptides that interact with HER2 protein based on the HER2/pertuzumab crystal structure (PDB entry: 1S78). *In silico* mutations were performed to screen for peptides with the lowest binding free energy, and OBOC peptide library screening was then carried out. Both the binding free energy calculation and the OBOC library screening found the peptide **58F63Y** to have the highest affinity to HER2. **58F63Y**, together with five other peptides, were selected for further analysis and experimental validation. All results show that the peptide **58F63Y** binds most favorably to HER2, with a dissociation constant (*K*_D_) of 536 nmol/L. The results of *ex vivo* and *in vivo* experiments using mouse xenografted tumors confirm that this peptide has strong affinity and high specificity to HER2. Binding free energy decomposition analysis [28–30] and distances calculation using Pymol found that there are more paired residues with low binding free energy and distances of less than 5 Å, which may explain the high affinity. Compared to other peptides that target HER2 [31], peptide **58F63Y** is unique in that it is acquired based on simulation using a different primary model with binding sites on domain II of the HER2 protein. Given its low toxicity, this peptide may be used as an alternative probe in the diagnosis and treatment of HER2-positive breast cancer and contributes to the HER2-targeting peptide library.

## Results/Discussion

### Structure analysis of the HER2/pertuzumab complex

**Fig 1:**
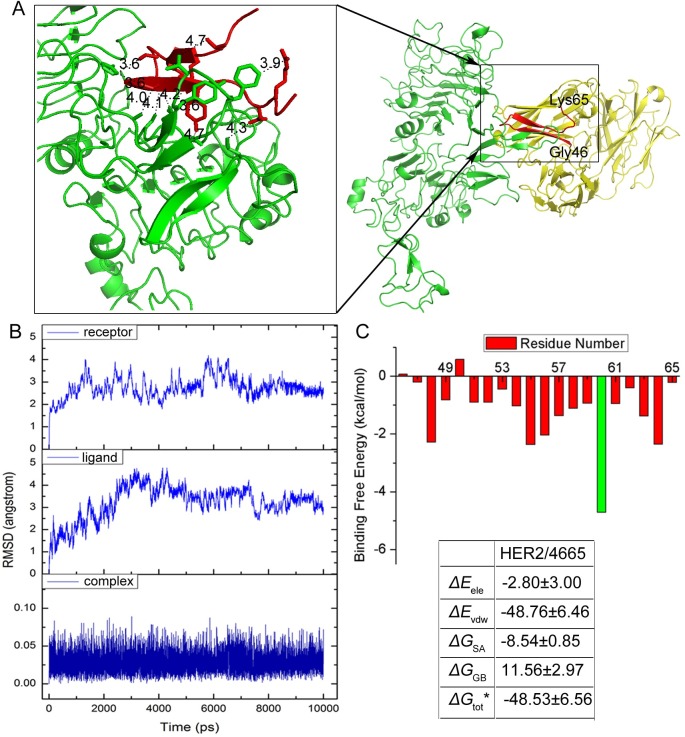
Analysis of HER2/pertuzumab complex. (A) Structure of the HER2 (green)/pertuzumab (yellow) complex. The fragment from Glu46 to Lys65 of pertuzumab is shown in red. The side chains that participate in key interactions are shown as sticks. Calculated distances (< 5 Å) are indicated with blue dash lines. (B) Backbone RMSDs as a function of time for the initial and successive structure of HER2/4665 complex in MD trajectories. **(**C) Binding free energies and individual energy terms for HER2/4665 and binding free energy decomposition for each residue of 4665. (* means that Δ*G*_tot_ does not contain -*TΔS* energy)

Pertuzumab is a monoclonal antibody marketed by Genentech for the treatment of HER2-positive breast cancer. Pertuzumab binds to HER2 at the center of domain II, sterically blocking the pocket essential to receptor dimerization and signaling. The HER2/pertuzumab crystal structure was obtained from the Protein Data Bank. In this structure, the soluble extracellular domain of HER2 [32] was crystallized in complex with the Fab fragment of the disulfide anti-HER2 monoclonal antibody pertuzumab [33]. Based on the calculation of distances for all residues between HER2 and pertuzumab and the selection of those within 5 Å, we found a peptide fragment of 20 residues in length (sequence: EWVADVNPNSGGSIYNQRFK) with a beta folding layer structure, named **4665**, that plays an important role in the interactions (Fig 1A). The HER2/fragment **4665** complex was chosen for further simulation analysis. MM/GBSA free energy was calculated based on 500 snapshots from 7 to 10 ns of MD simulation trajectories (Fig 1B) for each complex, as described in the Materials and methods section. The results show that the predicted binding free energy between **4665** and HER2 is –48.53 kcal/mol with the van der Waals (Δ*E*_vdw_) contribution being a main component.

### Mutations based on 4665

To improve the affinity of **4665** against HER2, we undertook single and double mutations and performed MD simulations to estimate the binding affinity. The properties of the interacting amino acids in both HER2 and peptides, as well as the space among the interactions, were considered. As shown in Fig 1C, Glu46, Trp47 and Lys65 have low energy contributions to the HER2/4665 complex. In addition, Asn52-Asn54 has almost no contributions to the binding. Therefore, mutations were made mostly in two beta strands in **4665**: one is from Val48 to Val51 with high hydrophobicity, and the other is from Ser55 to Phe64. The basic rules are that mutations should favor electrostatic and van der Waals interactions, and do not cause steric overlap. Briefly, the van der Waals (Δ*E*_vdw_) contribution is a main component in HER2/4665 interactions as suggested from the free energy calculation. As a consequence, residues in the strand of Ser55-Phe64 were preferably mutated to nonpolar amino acids (Ser55, Gly56, Gly57 and Gln62) to increase the van der Waals (Δ*E*_vdw_) contribution. Moreover, residues with large side chains are mutated into amino acids with similar side chain groups, such as Val48, Ala49, Gly56 and Gly57. We also took into account of the inter spaces between the peptide and HER2. For example, Ser58 is located on a beta strand that is close to the HER2 fragment Phe256-Lys314 but with a large spatial distance, so Ser58 was mutated to the residues with larger side chain groups or nonpolar amino acids. Arg63 is also located on a beta strand that is closer to the HER2 fragment Phe256-Lys314. Considering its distance (3.9 Å) to Phe257, Arg63 is mutated into residues with side chain groups no larger than benzol methyl or nonpolar residues. Finally, we first carried out 59 single mutations based on the **4665** sequence by performing MD simulations, and binding free energies were calculated for each mutant. Seventeen single mutations with binding free energies < –48.53 kcal/mol were selected to create combinations of double mutations, thus resulting in another 56 mutants. All mutations and their binding free energies are shown in Tables 1 and 2. Among these mutations, 34 sequences have lower binding free energies than the original **4665** peptide; peptide **58F63Y** (sequence: EWVADVNPNSGG**F**IYNQ**Y**FK) is the lowest, and so it is expected to bind most tightly to HER2.

**Table 1:** Binding free energies and individual energy terms for HER2 and 4665 (bold) or its single mutant peptide complexes calculated by MM/GBSA (kcal/mol).

	Δ*E*_ele_	Δ*E*_vdw_	Δ*G*_SA_	Δ*G*_GB_	Δ*G*_tot_^*^
**HER2/4665**	**-2.80±3.00**	**-48.76±6.46**	**-8.54±0.85**	**11.56±2.97**	**-48.53±6.56**
HER2/58F	-33.92±5.43	-61.32±5.64	-10.32±0.71	40.00±5.05	-65.55±5.95
HER2/55P	-12.73±2.62	-63.80±5.02	-8.65±0.36	20.74±2.47	-64.44±4.95
HER2/51Y	-21.91±13.75	-60.13±9.27	-10.39±1.48	31.15±13.62	-61.28±10.21
HER2/63Y	-2.62±7.34	-56.91±5.75	-9.91±0.79	9.74±6.67	-59.71±5.83
HER2/56M	-20.93±3.64	-57.53±4.35	-8.76±0.37	27.64±3.18	-59.58±4.31
HER2/63Q	3.57±4.23	-58.22±3.77	-8.57±0.36	3.83±4.00	-59.38±3.71
HER2/55M	-16.26±4.63	-57.40±4.92	-9.26±0.93	24.20±4.56	-58.72±4.77
HER2/64R	-55.95±5.13	-52.20±3.80	-8.03±0.38	59.76±4.73	-56.41±3.39
HER2/63A	-7.99±6.00	-54.06±5.57	-9.84±0.63	15.94±5.56	-55.95±5.22
HER2/59R	-57.39±4.30	-49.62±3.74	-8.08±0.45	60.92±4.19	-54.17±3.53
HER2/60W	-24.36±3.71	-50.18±4.32	-8.72±0.39	29.31±3.40	-53.95±4.03
HER2/62F	-24.79±8.48	-50.67±4.28	-8.13±0.63	29.68±7.73	-53.91±4.75
HER2/57A	-17.53±7.06	-50.83±7.18	-7.97±0.71	23.14±6.83	-53.18±7.83
HER2/55V	-14.15±9.94	-51.83±4.97	-8.19±0.76	21.13±9.66	-53.04±5.16
HER2/63W	-9.48±6.67	-50.27±5.80	-9.29±0.75	16.41±5.72	-52.63±5.68
HER2/58H	-17.03±4.45	-49.35±3.81	-7.91±0.36	22.31±3.94	-51.97±3.66
HER2/51H	-30.37±5.42	-47.35±3.89	-7.51±0.46	34.67±4.97	-50.56±4.04
HER2/63V	21.02±4.59	-51.23±5.14	-8.19±0.83	-11.28±4.21	-49.69±5.07
HER2/55A	-18.54±5.55	-46.71±2.99	-7.15±0.31	23.15±5.20	-49.24±2.85
HER2/56Y	-25.10±7.67	-45.58±3.57	-7.68±0.42	29.37±6.81	-49.00±3.67
HER2/57V	-11.49±6.49	-47.61±4.21	-7.65±0.78	17.78±6.26	-48.98±4.23
HER2/64Q	-4.94±3.74	-48.29±3.94	-7.04±0.40	11.66±3.51	-48.61±4.00
HER2/59M	-4.15±3.63	-47.45±5.88	-7.99±1.24	11.08±3.74	-48.51±5.58
HER2/50A	-38.99±8.54	-44.73±4.23	-7.44±0.57	42.83±8.20	-48.33±4.16
HER2/59Y	-18.24±4.71	-45.82±5.35	-7.29±0.51	24.18±4.36	-47.17±5.23
HER2/59L	-9.73±4.77	-43.83±4.52	-7.03±0.55	15.38±4.45	-45.20±4.44
HER2/62W	-12.61±3.38	-43.32±3.64	-7.16±0.45	18.79±3.36	-44.30±3.66
HER2/56F	-14.20±5.13	-43.20±6.33	-7.09±0.97	20.38±5.27	-44.12±6.75
HER2/64N	-9.10±3.10	-43.02±5.14	-6.98±0.68	15.24±2.97	-43.85±5.27
HER2/50R	-66.97±7.45	-39.17±4.30	-6.70±0.51	69.05±6.87	-43.79±4.49
HER2/56V	-11.03±4.32	-42.22±4.48	-7.48±0.67	17.26±4.07	-43.47±4.33
HER2/57L	-13.70±4.91	-40.82±4.03	-7.03±0.46	19.01±4.63	-42.55±4.00
HER2/50N	-39.06±6.24	-38.96±4.02	-7.07±0.43	42.91±5.73	-42.18±3.67
HER2/63F	12.94±5.89	-41.82±5.14	-7.00±0.74	-6.17±5.70	-42.05±4.97
HER2/64K	-52.42±5.63	-38.25±4.26	-7.00±0.49	55.69±4.92	-41.97±3.90
HER2/61Q	-10.87±7.04	-40.56±6.44	-6.87±1.15	16.36±6.78	-41.94±7.21
HER2/49M	-16.86±3.05	-40.93±3.81	-6.67±0.49	22.55±2.99	-41.90±3.90
HER2/49Y	-15.26±5.61	-39.89±3.62	-7.07±0.59	20.64±5.22	-41.58±3.57
HER2/55F	-7.42±3.88	-40.23±4.16	-5.84±0.81	12.05±4.11	-41.43±4.18
HER2/50Q	-31.24±2.89	-39.17±3.54	-6.05±0.41	35.46±2.68	-41.01±3.43
HER2/57M	-18.91±4.86	-37.70±3.65	-5.99±0.50	21.99±3.72	-40.62±2.90
HER2/55W	-16.78±3.25	-37.86±6.63	-6.77±0.60	20.99±2.84	-40.42±6.63
HER2/58M	-7.33±4.32	-39.24±5.57	-6.48±1.02	13.53±4.19	-39.52±5.66
HER2/48F	-12.35±6.48	-37.80±3.59	-6.70±0.47	17.41±5.99	-39.44±3.62
HER2/58Y	-9.84±3.45	-38.04±4.82	-6.33±0.71	15.17±3.20	-39.04±4.59
HER2/50K	-52.87±4.84	-35.59±3.71	-5.64±0.66	55.91±4.54	-38.20±4.16
HER2/50E	-1.73±4.67	-36.23±5.09	-6.29±0.54	7.86±4.46	-36.38±5.08
HER2/48W	-7.59±3.38	-35.36±7.43	-5.62±1.23	12.58±3.55	-35.99±8.18
HER2/63G	20.85±3.25	-35.39±3.81	-5.98±0.39	-15.06±2.92	-35.58±3.64
HER2/58W	-8.35±4.07	-34.16±7.03	-5.88±1.14	13.26±3.90	-35.13±7.18
HER2/50G	-46.10±8.84	-32.94±5.02	-5.44±0.71	49.85±8.81	-34.62±5.11
HER2/62Y	-5.51±2.47	-34.19±3.62	-4.80±0.51	10.02±2.32	-34.48±3.63
HER2/60F	-5.23±2.95	-33.57±3.67	-5.48±0.49	9.82±2.68	-34.45±3.53
HER2/48M	-14.11±6.56	-31.09±7.40	-6.12±1.33	18.77±6.78	-32.54±8.03
HER2/59K	-24.41±1.68	-30.60±2.14	-4.28±0.21	27.09±1.73	-32.20±2.08
HER2/62M	-1.45±2.02	-31.16±2.16	-4.33±0.21	5.41±1.98	-31.53±2.11
HER2/63M	18.31±1.67	-31.16±2.28	-4.28±0.30	-13.44±1.62	-30.58±2.20
HER2/55Y	-7.11±6.88	-27.50±12.12	-4.55±1.48	10.19±7.09	-28.97±12.80
HER2/48Y	-22.24±5.13	-25.21±5.00	-5.20±0.78	24.77±4.86	-27.86±5.05

Mutations are sorted with the lowest binding free energy on the top.

* means that Δ*G*_tot_ does not contain -*TΔS* energy.

**Table 2:** Binding free energies and individual energy terms for HER2 and 4665 (bold) or its double mutant peptide complexes calculated by MM/GBSA (kcal/mol).

	Δ*E*_ele_	Δ*E*_vdw_	Δ*G*_SA_	Δ*G*_GB_	Δ*G*_tot_^*^
**HER2/4665**	**-2.80±3.00**	**-48.76±6.46**	**-8.54±0.85**	**11.56±2.97**	**-48.53±6.56**
**HER2/58F63Y**	**-10.01±3.42**	**-63.71±5.35**	**-10.77±0.61**	**18.49±2.99**	**-65.99±5.29**
HER2/55V63Y	9.92±4.67	-64.75±3.94	-8.85±0.45	-1.34±4.05	-65.02±3.90
HER2/58H60W	-47.65±8.09	-56.14±5.69	-9.98±0.53	52.45±6.90	-61.32±5.53
HER2/55V63W	10.19±3.85	-60.31±4.62	-9.69±0.41	-0.61±3.36	-60.42±4.65
HER2/56Y63W	16.40±3.12	-59.88±4.69	-10.48±0.51	-6.34±2.80	-60.29±4.45
HER2/57A58H	-34.69±9.10	-55.49±4.14	-10.47±0.61	40.66±8.21	-59.99±4.10
HER2/56M60W	-23.37±6.10	-54.63±5.34	-8.29±0.64	27.77±5.66	-58.52±5.62
HER2/56M58F	-20.68±6.96	-56.75±5.24	-9.37±0.61	29.07±6.73	-57.73±5.22
HER2/58H63W	8.32±4.24	-56.54±4.19	-8.76±0.62	-0.45±3.58	-57.42±3.93
HER2/56Y57V	-20.81±3.68	-53.99±4.81	-8.59±0.40	26.92±3.32	-56.47±4.81
HER2/55M56M	-34.17±7.11	-52.10±6.11	-9.30±0.80	39.61±6.47	-55.95±6.45
HER2/57A63W	10.14±5.42	-53.94±4.99	-9.14±0.78	-0.23±4.73	-53.17±5.27
HER2/56M63W	13.60±5.03	-52.71±5.30	-8.29±0.61	-5.64±4.84	-53.04±5.63
HER2/56Y58H	-22.78±5.00	-49.78±5.30	-8.17±0.47	28.58±4.25	-52.16±4.78
HER2/60W63V	5.41±3.12	-49.94±4.17	-7.80±0.41	0.58±2.69	-51.75±4.13
HER2/55V57V	-14.89±6.48	-47.30±3.95	-8.01±0.43	19.66±5.82	-50.55±4.12
HER2/56M58H	-20.51±8.40	-47.44±3.91	-7.61±0.41	25.36±7.77	-50.20±4.34
HER2/57V60W	-12.76±5.78	-47.37±3.16	-7.42±0.36	17.83±5.54	-49.73±3.16
HER2/56Y58F	-13.35±5.80	-47.56±4.64	-7.50±0.46	19.12±4.97	-49.30±4.60
HER2/57A60W	-13.94±4.81	-46.98±4.14	-7.77±0.60	19.40±4.65	-49.29±4.37
HER2/60W63Y	7.83±4.20	-46.86±3.58	-7.55±0.35	-1.64±3.86	-48.23±3.52
HER2/58F63V	6.88±6.67	-46.34±3.28	-7.30±0.36	-1.21±6.26	-47.97±3.39
HER2/55M63V	14.18±4.17	-47.04±3.96	-6.95±0.43	-7.81±4.09	-47.63±4.00
HER2/55M57A	-9.15±8.00	-46.33±4.20	-7.44±0.56	15.67±7.38	-47.26±4.52
HER2/55V56Y	-3.38±3.67	-45.23±3.53	-6.83±0.44	8.76±3.49	-46.68±3.71
HER2/55V60W	-33.48±7.33	-41.96±6.81	-7.27±0.81	36.18±7.03	-46.53±6.88
HER2/57A58F	-29.17±9.26	-44.72±4.97	-8.88±0.52	36.61±7.88	-46.16±3.97
HER2/56Y57A	-18.39±7.91	-43.16±5.04	-7.47±0.71	23.01±7.17	-46.02±4.81
HER2/60W63W	12.75±3.09	-44.57±4.64	-7.17±0.54	-6.55±2.90	-45.54±4.98
HER2/57V63Y	17.24±6.77	-44.98±6.66	-7.88±0.78	-9.66±6.46	-45.28±6.31
HER2/56M57A	-12.93±5.31	-43.92±4.89	-7.46±0.91	19.48±5.73	-44.82±5.00
HER2/57V58H	-10.54±2.99	-41.86±3.85	-7.20±0.56	15.85±3.25	-43.75±3.84
HER2/56Y63V	10.06±5.47	-42.36±5.45	-7.16±0.79	-4.27±5.18	-43.74±5.97
HER2/55M63W	20.40±4.42	-42.91±3.68	-7.44±0.51	-13.07±4.30	-43.02±3.71
HER2/55V56M	-2.41±6.29	-41.48±4.58	-6.11±0.63	7.24±6.41	-42.77±4.48
HER2/55M56Y	-26.11±6.16	-39.13±4.40	-6.75±0.48	30.23±5.64	-41.75±4.33
HER2/56Y60W	-11.22±5.45	-39.93±4.29	-7.24±0.72	16.85±5.24	-41.55±4.29
HER2/56M57V	-13.94±6.10	-40.53±4.12	-7.26±0.70	20.23±5.81	-41.51±4.19
HER2/55V58F	-13.98±5.53	-38.96±5.07	-6.47±0.56	18.54±5.08	-40.87±4.71
HER2/57V58F	-20.38±5.57	-38.08±4.09	-7.07±0.44	25.14±4.66	-40.39±3.79
HER2/57A63V	20.03±3.49	-40.14±3.44	-6.67±0.38	-13.38±3.26	-40.16±3.42
HER2/55M63Y	14.18±3.60	-38.25±4.84	-6.12±0.62	-9.63±3.19	-39.83±4.69
HER2/58H63V	14.06±5.19	-39.22±4.34	-7.20±0.63	-6.95±4.67	-39.31±4.35
HER2/58F63W	12.75±3.12	-38.60±3.15	-6.84±0.39	-6.29±3.08	-38.98±3.14
HER2/55M57V	-9.85±5.40	-35.73±7.07	-5.96±1.00	14.22±5.73	-37.32±7.47
HER2/58F60W	-10.95±3.71	-34.78±4.21	-6.61±0.55	15.66±3.43	-36.68±3.97
HER2/55V58H	-20.42±4.43	-32.39±4.23	-5.78±0.71	23.85±4.17	-34.74±4.51
HER2/56M63V	18.42±2.43	-35.37±3.04	-6.58±0.44	-11.19±1.95	-34.72±2.80
HER2/55M58F	0.30±4.01	-33.65±4.03	-5.21±0.84	4.30±4.42	-34.26±3.80
HER2/55V57A	-5.08±3.63	-34.08±4.04	-4.66±0.63	10.03±3.91	-33.79±3.69
HER2/57V63W	13.14±5.88	-33.28±5.81	-5.71±1.08	-7.10±5.63	-32.95±6.60
HER2/58H63Y	22.87±2.16	-31.54±2.39	-4.23±0.18	-17.85±2.09	-30.74±2.38
HER2/55V63V	22.36±6.00	-32.34±3.88	-6.35±0.69	-14.00±6.12	-30.33±4.09
HER2/56M63Y	15.13±1.96	-29.23±5.46	-4.41±0.83	-9.73±2.50	-28.24±5.27
HER2/57V63V	13.57±2.00	-29.01±3.20	-4.26±0.44	-8.09±1.90	-27.79±3.01
HER2/57A63Y	17.11±4.40	-24.90±6.45	-4.81±0.99	-11.59±4.73	-24.19±6.67

Mutations are listed with those bearing the lowest binding free energy near the top; 58F63Y (bold) was selected for further analysis.

* means that Δ*G*_tot_ does not contain -*TΔS* energy.

### OBOC peptide library screening

**Fig 2:**
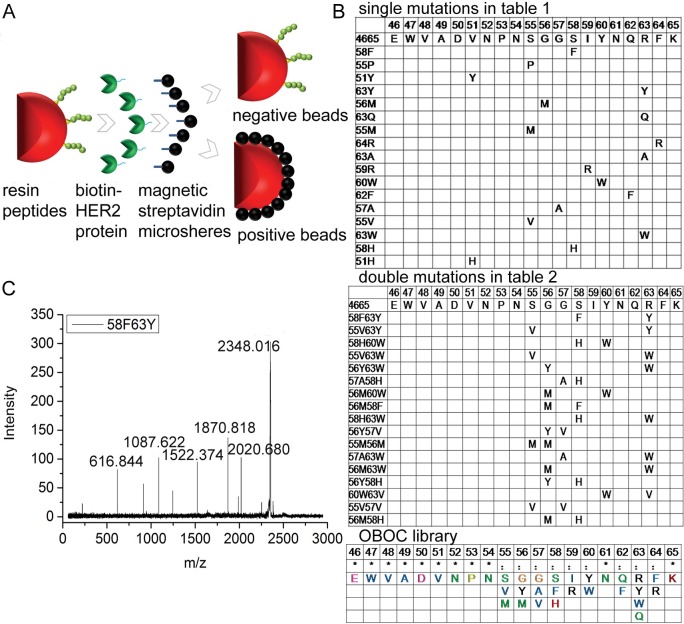
OBOC peptide library screening. A. A sketch of the method. B. Selected amino acids for mutation in each position based on MD simulations. C. MS/MS peaks of peptide **58F63Y**.

The results of our previous work show that when receptor–ligand interactions are similar—save for only a few residue differences—computational binding free energy calculations can closely reflect the relative affinity of peptide binding [31,34]. In the current study, to determine whether the above *in silico* screening correctly identified a peptide with affinity among the highest ones, the OBOC peptide library approach (Fig 2A) was later performed. We designed the peptide library based on the calculated binding free energies of peptides/HER2 with single and double mutations, using MD simulations (Tables 1 and 2). Mutations with binding free energies lower than the wild type **4665** (Δ*G*_tot_ < –50 kcal/mol) were selected, and the intersection of single and double mutations from 55 to 64 was used in library construction (Fig 2B), resulting in a library of 5184 sequences. Biological screening of the OBOC peptide library is routinely carried out as described in the Materials and methods section. Three positive beads were identified, and following MALDI–TOF–MS/MS analysis, one of them was found to be the same as the peptide **58F63Y**, which has the lowest binding free energy with MD simulation (Fig 2C).

### Dissociation constants of peptides measured by surface plasmon resonance imaging

**Fig 3:**
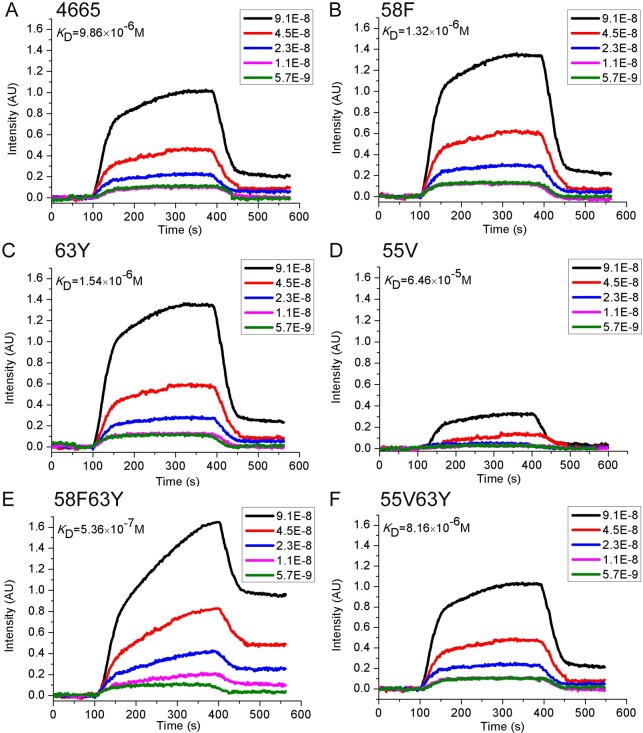
SPRi detection of the binding affinity of peptides. 4665 (A), 58F (B), 63Y (C), 55V (D), 58F63Y (E), and 55V63Y (F) towards HER2. The dissociation constant was calculated from kinetic constants obtained by fitting association and dissociation curves to real-time binding and washing data. 58F63Y has the highest affinity of all six peptides.

After combining the results, peptide **58F63Y** was selected for further experimental validation. Another double mutant **55V63Y** and its single mutations **58F**, **63Y**, and **55V**—as well as the original wild type **4665**—were also selected to facilitate better comparison. The sequences of these six peptides were aligned using Clustal Omega; the results are shown in S1 Fig. Surface plasmon resonance imaging (SPRi)—which has been previously used to estimate interactions between molecules for the purposes of disease diagnosis, drug discovery, and peptide screening [23,24,35–37]—was used in this study to estimate the dissociation constants of peptides binding to HER2, as described in the Materials and methods section. The dissociation constant was calculated from kinetic constants obtained by fitting association and dissociation curves to real-time binding and washing data. While none of the four peptides shows any affinity to the HSA protein (S2 Fig), Fig 3 indicates that the *K*_D_ values of the peptides **4665**, **58F**, **63Y**, **55V**, **58F63Y**, and **55V63Y** with HER2 protein are 9.86 μmol/L, 1.32 μmol/L, 1.54 μmol/L, 64.6 μmol/L, 0.536 μmol/L, and 8.16 μmol/L, respectively. We can see that, to some extent, the *K*_D_ values agree with the binding free energies from the simulation, which range from –48 kcal/mol to –66 kcal/mol. This finding is consistent with that of our previous work, in which it was found that computational binding free energy from MM/GBSA can be used to estimate the relative affinity of peptide binding. Based on the SPRi results, three peptides (**58F**, **63Y**, **58F63Y**) with the highest affinity, as well as the starting peptide **4665**, were chosen for later confocal fluorescence imaging analyses.

### Validation of affinity and specificity of peptides to HER2 high expression cells

**Fig 4:**
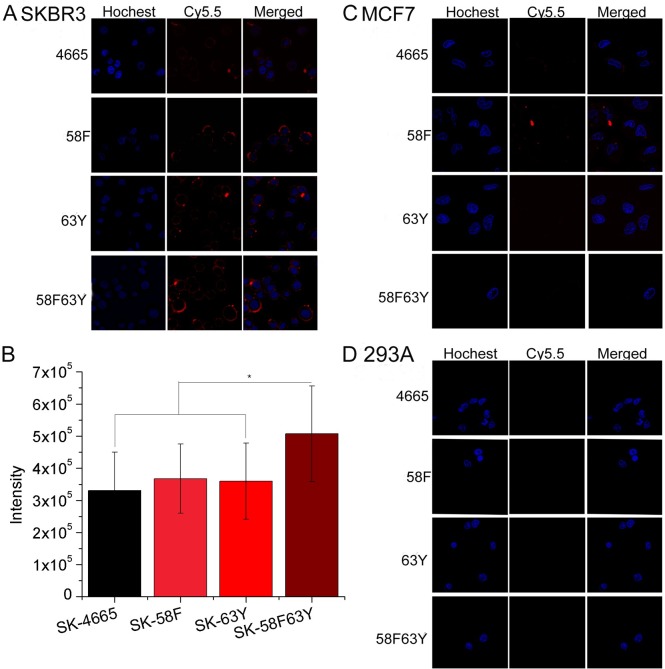
Confocal fluorescence imaging analysis of cells incubated with Cy5.5-labeled peptides. (A) SKBR3 cell line with high HER2 expression, (B) Quantified intensities of Cy5.5-peptides in SKBR3 cell line, (C) MCF cell line with medium HER2 expression, and (D) 293A cell line with no HER2 expression. Cy5.5-labeled peptide is shown in red, and Hoechst in blue.

Four tumor cell lines (SKBR3, MCF7, MDA-MB-468, and 293A), each with a different HER2 expression level, were used in confocal fluorescence imaging analysis to confirm the binding specificity of peptides to the HER2 protein. Among these cell lines, the expression of HER2 was found to be high in SKBR3, medium in MCF7, and low in MDA-MB-468 and in 293A. As shown in Fig 4 and S3 Fig, when treated with Cy5.5-peptides, fluorescent intensities are strong in SKBR3 cells, but weaker in MCF7 cells and absent in MDA-MB-468 and 293A cells. Especially, SKBR3 cells treated with Cy5.5-58F63Y show the strongest fluorescence, thus indicating that **58F63Y** binds with the highest affinity to HER2 (Fig 4B); this finding is consistent with the aforementioned MD calculation and SPRi analytical results. All these results confirm that peptides can specifically bind at the cellular level to the extracellular domain of the HER2 protein. Peptide **58F63Y** (which had the highest affinity) and the wild type **4665** were chosen for subsequent *in vivo* studies. Toxicity of peptides **58F63Y and 4665** to HUVEC and SKBR3 cells was also measured**. 4665** and **58F63Y** shows no toxicity to both cell lines (S4 Fig).

### Confirmation of affinity and specificity of peptides to HER2-positive tumors

**Fig 5:**
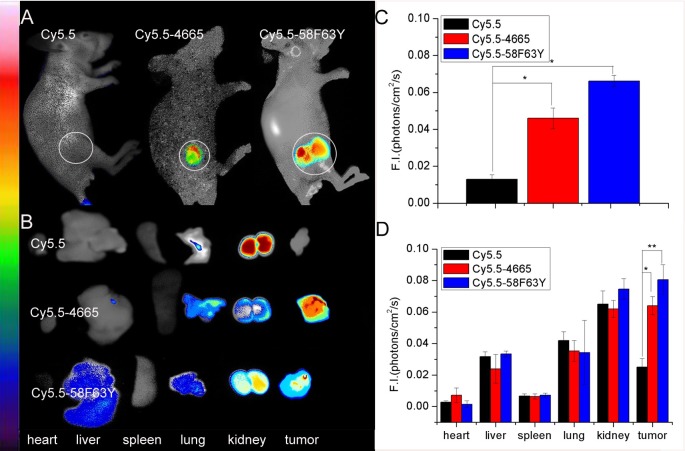
*In vivo* and *ex vivo* imaging of tumor targeting by 4665 and 58F63Y. (A) *in vivo* fluorescence imaging of 4665 and 58F63Y to tumor, (B) *ex vivo* fluorescence imaging of tumor accumulation and biodistribution, and (C, D) quantification of the fluorescence signals *in vivo* and *ex vivo*, respectively. Fluorescence intensity was measured in terms of counts/energy/area and presented as an average (n = 3).

To investigate the affinity and specificity of peptides to HER2-positive tumors *in vivo*, nude mice bearing subcutaneous SKBR3 tumor xenografts were intravenously injected with Cy5.5-labeled peptides and Cy5.5 as the control; they were then subjected to whole-body optical imaging, using a small animal *in vivo* imaging system (CRI Maestro 2). Fig 5A shows clear differences between the tumor images of mice with Cy5.5–58F63Y or Cy5.5–4665 and those of the control mice. The intensities are plotted in bar charts (Fig 5C) that indicate that binding affinity increases 5.09-fold for **58F63Y** and 3.52-fold for **4665**, relative to the control. Moreover, fluorescence images of the dissected organs of the experimental mice, taken 30 min postinjection with Cy5.5-labeled peptides or control Cy5.5, were acquired for further examination. Both images and quantification in the bar charts (Fig 5B and 5D) show that tumors treated with **58F63Y** and **4665** have significantly high fluorescence signals, compared to those in the controls. Among all the organs, the kidney was found to have the highest background signal for both peptides and control, probably due to the toxic effect of Cy5.5 [38,39]. Taken together, all the results demonstrate that **58F63Y** has high specificity and affinity for HER2-positive tumors.

### *In silico* analysis of interactions between peptides and HER2

**Fig 6:**
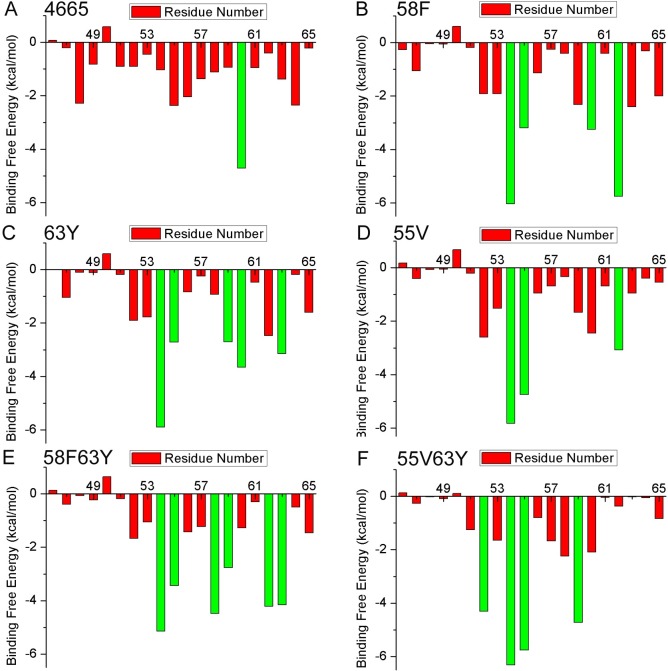
Binding free energy decomposition for each residue of the ligands in the six complexes. HER2/4665 (A), HER2/58F (B), HER2/63Y (C), HER2/55V (D), HER2/58F63Y (E), and HER2/55V63Y (F). The green bars in the figure represent a binding free energy < –2.5 kcal/mol. Each mutant from B to F has more than three residues that are favorable to HER2 binding.

**Fig 7:**
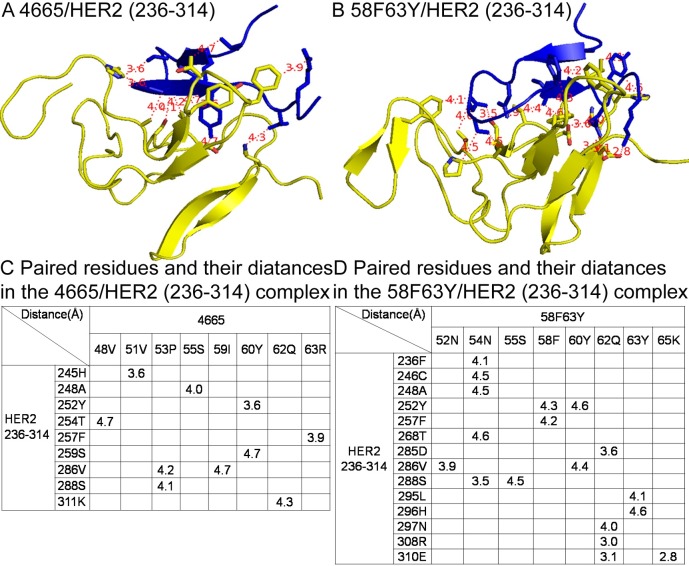
Paired residues in HER2/peptide complexes. (A) 4665/HER2 fragment 236-314; (B) 58F63Y/HER2 fragment 236-314; (C) Paired residues within 5 Å and their distances in the 4665/HER2 and (D) 58F63Y/HER2 complexes. Blue cartoons stand for the mutant peptides; yellow cartoons for the fragment 236 to 314 in the HER2 protein; and red dashed lines show paired residues within 5 Å.

To better understand the reason of the high affinity of **58F63Y** to HER2, a more detailed *in silico* analysis was performed. Another four mutants—as well as the original wild type **4665**—were also selected for a more comprehensive comparison. To verify peptide structure stability after binding to HER2, the root mean square deviation (RMSD) values of the backbone atoms of the initial structure and of successive simulated structures were calculated for all six HER2/peptides complexes. S5 Fig shows that for all six complexes, the RMSD values become stable after about 5000 ps in the MD trajectories, thus indicating the convergence of each peptide and the complex structures towards an equilibrium state. A glance at the results of the free energy decomposition analysis of the peptides found that more residues have low energies in the mutants than in **4665**. Specifically, for **4665**, except Tyr60, no other residue contributes energy < –2.5 kcal/mol (Fig 6A). However, in the mutants (Fig 6B–6F)—besides Asn54 and Ser55 in all mutants—the following were found to contribute energy < –2.5 kcal/mol: Tyr60 and Gln62 in **58F**; Ser58, Ile59, and Tyr63 in **63Y**; Gln62 in **55V**; Ser58, Phe59, Gln62, and Arg63 in **58F63Y**; and Asn52 and Ile59 in **55V63Y**. That is to say, each mutant has more than three residues that are favorable to HER2 binding. From S6 Fig, we can see that all six peptides overlay in the same pocket of the HER2 protein, thus indicating that the binding sites of these peptides remain the same as those in the wild type. MM/GBSA binding free energy calculation and decomposition analysis of the HER2 protein reveals that fragment 236–314 has major interactions with these peptides, and that for each mutant, more than three residues have binding free energies < –3 kcal/mol (S7 Fig). Together, the beneficial changes of each mutant contribute to a lower binding free energy than that in wild type **4665**. Models from PyMOL were used to better visualize the key interacting residues in peptides/HER2 complexes (S8 Fig). In Fig 7, 18 residues pairs have distances of less than 5 Å in 58F63Y**/**HER2 (236-314), compared with 10 pairs in 4665**/**HER2 (236-314). As a summary, more residues pairs with lower binding free energy and close distances in the 58F63Y/HER2 complex may contribute to the high affinity of **58F63Y**.

In summary, based on the crystal structure of HER2/pertuzumab, we acquired a peptide that was 20 residues in length (i.e., **58F63Y**) that targets the HER2 protein; these findings were derived through the use of mutations and the computational calculation of the affinity with a combination protocol of molecular dynamics modeling, MM/GBSA binding free energy calculations, as well as the screening of an OBOC peptide library based on the mutations from the *in silico* modeling. This work proves that MM/GBSA binding free energy can be used to reflect the relative affinity of peptide binding closely. The peptide **58F63Y** has a *K*_D_ value of 536 nmol/L and binds to HER2 at the same site as the parent fragment of pertuzumab. Through confocal fluorescence imaging and *in vivo* and *ex vivo* studies, the peptide was found to have high affinity and specificity for the extracellular domain of HER2. We expect this peptide to serve as an alternative probe that can be used in combination with others to improve the early detection, diagnosis, and targeted therapy of HER2-positive breast cancer.

## Methods

### Prepare the initial structure

The primary sequences of the pertuzumab fragment from 46 to 65 (named **4665**) and its mutants **58F**, **63Y**, **55V**, **58F63Y**, and **55V63Y** were aligned by using the Clustal Omega program, which is available on EMBnet website (http://www.ebi.ac.uk/Tools/msa/clustalo/). The model for the HER2/4665 complex derives from the crystal structure of the HER2 extracellular region and pertuzumab in the RCSB PDB. The model for HER2/4665 was constructed based on the crystal structure of HER2/pertuzumab, by keeping related amino acids in pertuzumab. Other models were constructed based on HER2/4665.

### MD simulations

The AMBER03 force field was used to investigate the potentials of the complexes in the following molecular mechanics minimizations and MD simulations [40]. Missing atoms were added by using the *tleap* program. The whole system was solvated with TIP3P [41] water molecules in a truncated octahedron box with a minimum solute box-edge distance of 12 Å [42]. Then, the largest negative coulombic potential around the protein was randomly neutralized with counter-ions Na^+^ placed on the grids. The numbers of water molecules and Na^+^ ions in each system are listed in the supporting information (S1 Table). To remove poor-quality contacts between the complex and the solvent molecules, three-step energy minimization was performed by using the *sander* module of AMBER12 prior to undertaking the MD simulations. First, the whole protein was fixed and the water molecules and counter-ions were minimized; second, the backbone atoms of the protein were fixed and the side chains were minimized using the same settings as above; third, the whole system was minimized without any constraints. The first two stages consisted of a 5,000-cycle steepest descent and a 2,500-cycle conjugate gradient minimization; the final step consisted of 10,000 cycles of steepest descent and 5,000 cycles of conjugate gradient minimization. The SHAKE [43] method was applied to constrain covalent bonds related to hydrogen atoms, with a tolerance of 10^−5^ Å. Particle Mesh Ewald [44] was employed to adequately deal with long-range electrostatic interactions, and in the MD simulations, the cutoff distances for nonbond energy interactions were set to 12 Å [45]. Then, the entire system was gradually heated from 0 to 310 K in seven steps [46,47] over 60 ps [46,48] in the NVT (canonical ensemble). Finally, 10 ns MD simulations were implemented with a 2 fs [49] time step under the constant temperature of 310 K. During the sampling process, the trajectories were saved every 0.2 ps, and the conformations generated from the simulations were used in further analysis.

### Binding free energy calculations

MM/GBSA [50] serves as an effective computational tool in analyzing biomolecular interactions. When used with knowledge-based energy terms, MM/GBSA can help determine the binding free energies of all systems, based on the calculation of the average free energies of solvation (Δ*G*_bind_) between targeted protein and ligands over trajectories of MD simulation. The MM/GBSA method can be summarized as the following equation.

$$\begin{array}{l} \Delta G_{\mathrm{bind}}=G_{\mathrm{complex}}–G_{\mathrm{protein}}–G_{\mathrm{ligand}}\\ \;=\Delta E_{\mathrm{MM}}+\Delta G_{\mathrm{GB}}+\Delta G_{\mathrm{SA}}–T\Delta S\\ \;=\Delta E_{\mathrm{vdw}}+\Delta E_{\mathrm{ele}}+\Delta G_{\mathrm{GB}}+\Delta G_{\mathrm{SA}}–T\Delta S, \end{array}$$ (1)

In which, Δ*G*_bind_ represents the binding free energy in solution consisting of the molecular mechanics free energy (Δ*E*_MM_), the conformational entropic effect to binding (−*T*Δ*S*) in the gas phase, and the solvation free energy containing polar contribution (Δ*G*_GB_) and nonpolar contribution (Δ*G*_SA_). The Δ*E*_MM_ term includes Δ*E*_ele_ (electrostatic) and Δ*E*_vdw_ (van der Waals) energies and was calculated by the *sander* module of AMBER12. The polar contribution was calculated by using the GB [51] mode, with solvent and the solute dielectric constants set to 80 and 4, respectively. Additionally, the nonpolar energy was estimated, with a solvent-probe radius of 1.4 Å: Δ*G*_SA_ = 0.0072 × ΔSASA [52], by the LCPO method [50] based on the SASA model [53]. For each ligand–protein, 500 snapshots were taken from 7 to 10 ns on the MD trajectories. Due to the low prediction accuracy and the high computational cost [54,55] upon the nmode module in AMBER12 as well as their similar values in analogical system [31,34], the entropic contribution was ignored in the calculation of the predicted total binding free energy (Δ*G*_tot_^*^ means that Δ*G*_tot_ does not contain -*TΔS* energy).

### Free energy decomposition analysis

The specific inhibitor-residue interaction spectra were generated by using MM/GBSA decomposition analysis [28,56] undertaken through the *mm_pbsa* program of AMBER12. Four kinds of energy were found—namely, Δ*E*_vdw_, Δ*E*_ele_, Δ*G*_GB_, and Δ*G*_SA_—and each contributed to the binding interaction of each ligand–residue pair. The Δ*E*_vdw_ and Δ*E*_ele_ energy terms were calculated by the *sander* module of AMBER12. The polar contribution (Δ*G*_GB_) to solvation energy was calculated by using the GB module and the parameters for the GB calculation were developed by Onufriev et al. [57]. The nonpolar solvation contribution (Δ*G*_SA_) part was computed based on the SASA determined through the ICOSA method [52]. All energy components were calculated by using 500 snapshots extracted from the last 3 ns of the MD trajectories. After undertaking the decomposition process, the free energy contribution could be allocated to each residue from the association between the receptor and the ligand. Graphic visualizations and presentations of protein structures were generated by using PyMOL [58–60].

### Magnetic beads screening from peptide library

The OBOC peptide library was designed based on binding free energies derived from the MD simulations of single and double mutants that are lower than the HER2/4665 complex. These mutations are in the 4665 fragment of pertuzumab from 55 to 64: EWVADVNPNX_55_X_56_X_57_X_58_X_59_X_60_N_61_X_62_X_63_X_64_K. According to computational calculations, the V, S, and M mutants have lower energies for X_55_. This is also the case for G, M, and Y for X_56_; G, A, and V for X_57_; S, F, and H for X_58_; I and R for X_59_; Y and W for X_60_; Q and F for X_62_; R, W, Q, and V for X_63_; and F and R for X_64_. The result is a 3*3*3*3*2*2*2*4*2 = 5184 library capacity.

The OBOC library synthesis and screening was performed as per the previously used method [61–64]. Briefly, HBTU (4 mmol) and Fmoc-amino acid (4 mmol) reagent was dissolved in 0.4 mol/L N-Methyl morpholine in N,N-dimethylformamide and coupled with the solid phase supporting materials for 40 min during the coupling step. A 20% piperidine was used to remove the Fmoc group for 10 min in the deprotection step. During the OBOC library synthesis, the amino acid coupling process was carried out in the “split” step, while the deprotection process was carried out in the “pool” step. After elongation, a trifluoroacetic acid cleavage reagent was introduced to cleave the side chain protection group of each residue. Afterwards, the solid phase supporting materials were incubated with 5% milk, then with HER2/biotin complex, and then with monodispersed magnetic streptavidin microspheres. Each step was performed in an incubator at 37°C for 2 h, and followed by three washes with PBS. The HER2 protein was biotinylated using a biotinylation kit (Solulink Inc., USA). Positive beads with dark colors were picked out for the *in situ* chemical cleavage before MALDI–TOF–MS/MS analysis, and a 30 mg/mL cyanogen bromide solution was used overnight.

### Peptide synthesis

Peptides were synthesized using Fmoc strategy solid phase peptide synthesis [65–67]. Unsophisticated peptides were purified using a Hitachi HPLC system (L-7100, Japan) on a TSK gel ODS-100V reversed-phase column. Peptides were eluted with a linear gradient of 5–80% acetonitrile containing 0.1% trifluoroacetic acid at a flow rate of 2 mL/min within 25 min. Peptides were then subjected to MALDI–TOF–MS (Bruker Daltonics) analysis. Purified peptides were dried in vacuum desiccators and then stored at –20°C until further use.

2-(1H-benzotriazole-1-yl)-1, 1, 3, 3-tetramethyluronium hexafluorophosphate was purchased from GL Biochem (China). Trifluoroacetic acid and fluorescein 5-isothiocyanate were acquired from Sigma-Aldrich Co. LLC (USA). N-Methyl morpholine and N, N-dimethylformamide were acquired from a Beijing chemical plant (China).

### SPRi assay

For SPRi analysis, a cysteine residue linked to the amino terminal of all peptides was used for interacting with a bare gold chip bearing a 47.5-nm thickness. First, 1 μL peptides at 1 mg/mL was added to the gold surface of the chip and incubated overnight at 4°C. The chip was then washed with PBS and deionized water three times, and 5% nonfat milk was applied to block overnight at 4°C. After the chip was washed again with PBS and water, it was dried with nitrogen for later use. Human serum albumin (HSA) protein (Sigma-Aldrich Co. LLC) was used as the control. HER2 (Sino Biological Inc., China) and HSA proteins (Sigma-Aldrich Co. LLC) were dissolved in PBST and diluted to 10, 5, 2.5, 1.25, or 0.625 μg/mL. The SPRi analytical procedure was carried out on the prepared SPRi chip by running PBST buffer for baseline stabilization, followed by the protein sample, a PBST running buffer for washing, and finally 0.5% H_3_PO_4_ in deionized water for regeneration. This cycle was repeated for each concentration of HER2 and HSA protein at 20, 10, 5, 2.5, 1.25, and 0.625 μg/mL. Real-time binding signals were recorded and analyzed through the use of a PlexArray HT system (Plexera LLC, Bothell, WA, USA). The dissociation constant was calculated by fitting the association–dissociation curves.

### Immunocytochemistry

Four cell lines (SKBR3, MCF7, MDA-MB-468, and 293A) were seeded at a density of 3000 cells/mL into culture dishes and allowed to culture overnight with 5% CO_2_ at 37°C. Cell nuclei was stained with 1 mM Hoechst 33342 in 200 μL cell culture medium and incubated at 37°C for 15 min. Then, cells were incubated in culture medium with 50 μM Cy5.5-labeled peptide at 4°C for 20 min. Finally, cells were washed three times with cold PBS for observation. An Olympus FV1000-IX81 confocal-laser scanning microscope was used for confocal fluorescence imaging. An FV5-LAMAR 633 nm laser was used as the excitation source, and the emission wave length was collected at 690 nm. Hoechst 33342 was excited by a FV5-LD405-2 405 nm laser and collected within the range of 422–472 nm. All microscope parameters were identical for all observations of the binding ability of the various peptides.

### Ethics statement

All animal experiments were conducted in compliance with the Beijing University Animal Study Committee’s requirements vis-à-vis the care and use of laboratory animals. The Beijing University Animal Study Committee approved the experimental protocols.

### *In vivo* and *ex vivo* fluorescence imaging

Five to six-week-old Balb/c female nude mice were subcutaneously administered approximately 1 × 10^7^ SKBR3 cells into the right hind leg, to establish xenografted tumors. Thereafter, tumor size was periodically measured with a caliper, and mice with tumors of 6−8 mm in diameter were selected for the following small animal experiments. Cy5.5–NHS (Lumiprobe) was used to label peptides. Either Cy5.5-peptides or the control Cy5.5 (1 μM, 200 μL) was intravenously injected into tumor-bearing nude mice via the tail vein. The mice were anesthetized and fluorescence signals measured using the small animal *in vivo* imaging system 30 min postinjection. Three mice were used for each peptide and for control. Near-infrared fluorescence imaging of tumor-bearing nude mice were taken with an exposure time of 50 ms, using the Cy5.5 filter sets (excitation: 673 nm; emission: 707 nm), and the intensities were quantified using the same software. Then, fluorescence images of the main organs and of tumors dissected from nude mice were individually taken as above.

## Supporting information

S1 Fig Alignment of the sequences of the wild type and the five mutant peptides by Clustal Omega. (PDF)

S2 Fig SPRi analysis of the binding affinity of peptides. 4665 (A), 58F (B), 63Y (C), 55V (D), 58F63Y (E), and 55V63Y (F) toward HSA protein. The dissociation constant was calculated from the kinetic constants obtained by fitting the association and dissociation curves to the real-time binding and washing data.
(PDF)

S3 Fig Confocal fluorescence imaging analysis of the MDA-MB-468 cell line with low HER2 expression after incubating with peptides labeled with Cy5.5. (PDF)

S4 Fig **Toxicity of the peptides to HUVEC (A) and SKBR3 (B).** 4665 and 58F63Y shows no toxicity to both cell lines.
(PDF)

S5 Fig Backbone RMSDs as a function of time for the initial and successive structures of HER2/peptides complexes in MD trajectories. (A) HER2/4665, (B) HER2/58F, (C) HER2/63Y, (D) HER2/55V, (E) HER2/58F63Y, and (F) HER2/55V63Y. The RMSD values become stable after about 5000ps in the MD trajectories, indicating the convergence of each peptide structure towards an equilibrium state.
(PDF)

S6 Fig Comparison of the interactions of HER2/peptides. HER2/4665 (A), HER2/58F (B), HER2/63Y (C), HER2/55V (D), HER2/58F63Y (E), and HER2/55V63Y (F). Gray cartoons are the HER2 protein, and blue cartoons are the mutant peptides. Orange spots show the main contributing residues of each peptide interacting with HER2 protein.
(PDF)

S7 Fig Binding free energy decomposition for key residues of the HER2 protein in the six complexes. HER2/4665 (A), HER2/58F (B), HER2/63Y (C), HER2/55V (D), HER2/58F63Y (E), and HER2/55V63Y (F). Green bars in the figure represent binding free energy <–3 kcal/mol. Therefore, each mutant shown in B to F has more than three residues that contribute to the HER2/peptide complexes.
(PDF)

S8 Fig Binding models of HER2/peptides complexes. 4665/HER2 (A), 58F/HER2 (B), 63Y4665/HER2 (C), 55V/HER2 (D), 58F63Y/HER2 (E), and 55V63Y/HER2 (F). Blue cartoons stand for mutant peptides. Key residues (binding free energy <–1 kcal/mol) in HER2 protein are shown as yellow sticks.
(PDF)

S1 Table Numbers of water molecules and Na+ ions added in all simulation systems. (PDF)
