# Supplementary material for: Peptide probes derived from pertuzumab by molecular dynamics modeling for HER2 positive tumor imaging
Source: PLoS Comput Biol. 2017 Apr 13;13(4):e1005441. doi: 10.1371/journal.pcbi.1005441 (PMC5390981; doi:10.1371/journal.pcbi.1005441)
Supplement: S1 Fig — (PDF) [file pcbi.1005441.s001.pdf]

4665	EWVADVNPNSGGSIYNQRFK
58F	EWVADVNPNSGGFIYNQRFK
63Y	EWVADVNPNSGGSIYNQYFK
55V	EWVADVNPNVGGSIYNQRFK
58F63Y	EWVADVNPNSGGFIYNQYFK
55V63Y	EWVADVNPNVGGSIYNQYFK

S1 Fig. Alignment of the sequences of the wild type and the five mutant peptides by Clustal Omega.
