# Supplementary material for: Peptide probes derived from pertuzumab by molecular dynamics modeling for HER2 positive tumor imaging
Source: PLoS Comput Biol. 2017 Apr 13;13(4):e1005441. doi: 10.1371/journal.pcbi.1005441 (PMC5390981; doi:10.1371/journal.pcbi.1005441)
Supplement: S2 Fig — 4665 (A), 58F (B), 63Y (C), 55V (D), 58F63Y (E), and 55V63Y (F) toward HSA protein. The dissociation constant was calculated from the kinetic constants obtained by fitting the association and dissociation curves to the real-time binding and washing data. (PDF) [file pcbi.1005441.s002.pdf]

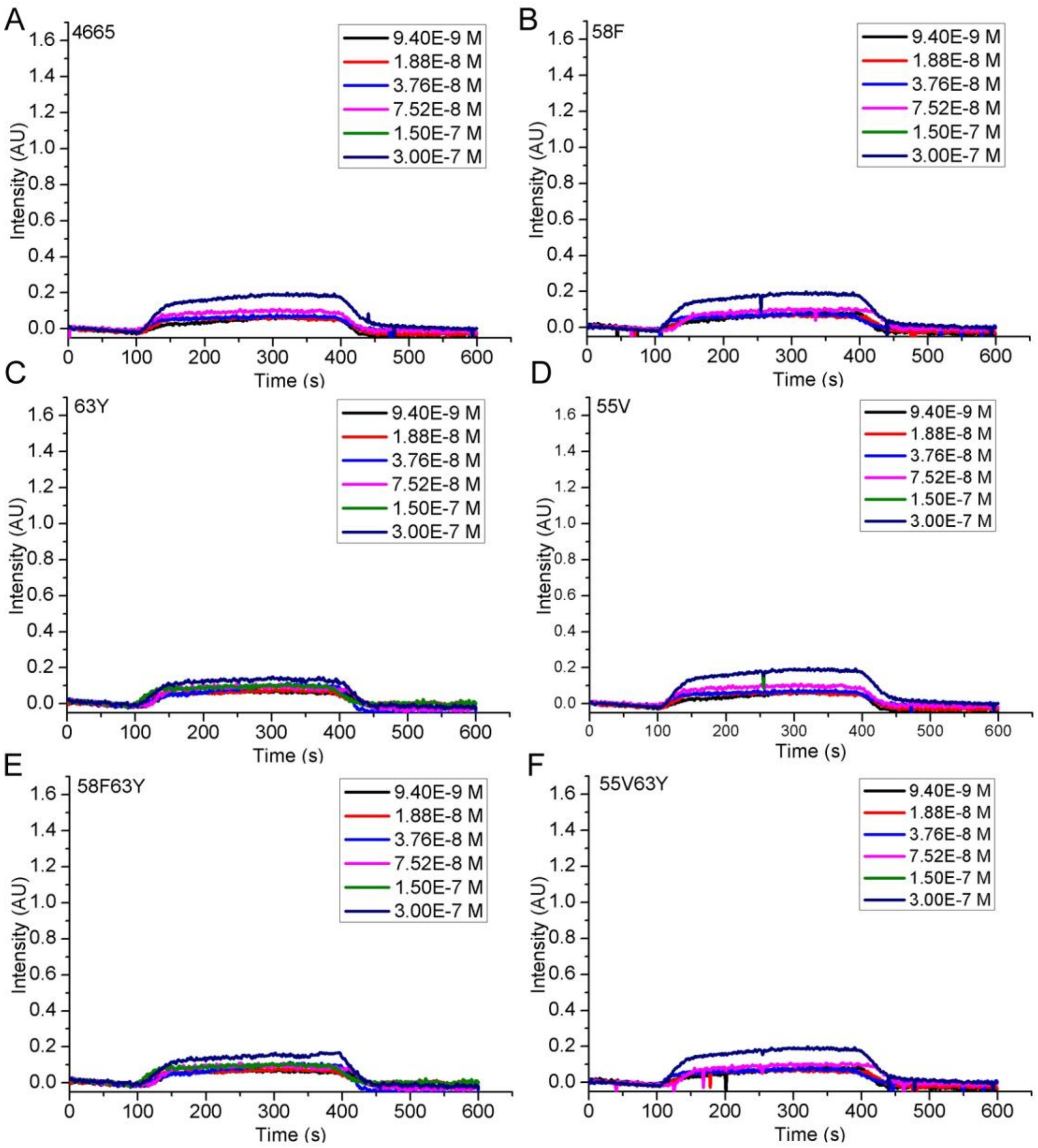

S2 Fig. SPRi analysis of the binding affinity of peptides: 4665 (A), 58F (B), 63Y (C), 55V (D), 58F63Y (E), and 55V63Y (F) toward HSA protein. The dissociation constant was calculated from the kinetic constants obtained by fitting the association and dissociation curves to the real-time binding and washing data.
