# Supplementary material for: Peptide probes derived from pertuzumab by molecular dynamics modeling for HER2 positive tumor imaging
Source: PLoS Comput Biol. 2017 Apr 13;13(4):e1005441. doi: 10.1371/journal.pcbi.1005441 (PMC5390981; doi:10.1371/journal.pcbi.1005441)
Supplement: S3 Fig — (PDF) [file pcbi.1005441.s003.pdf]

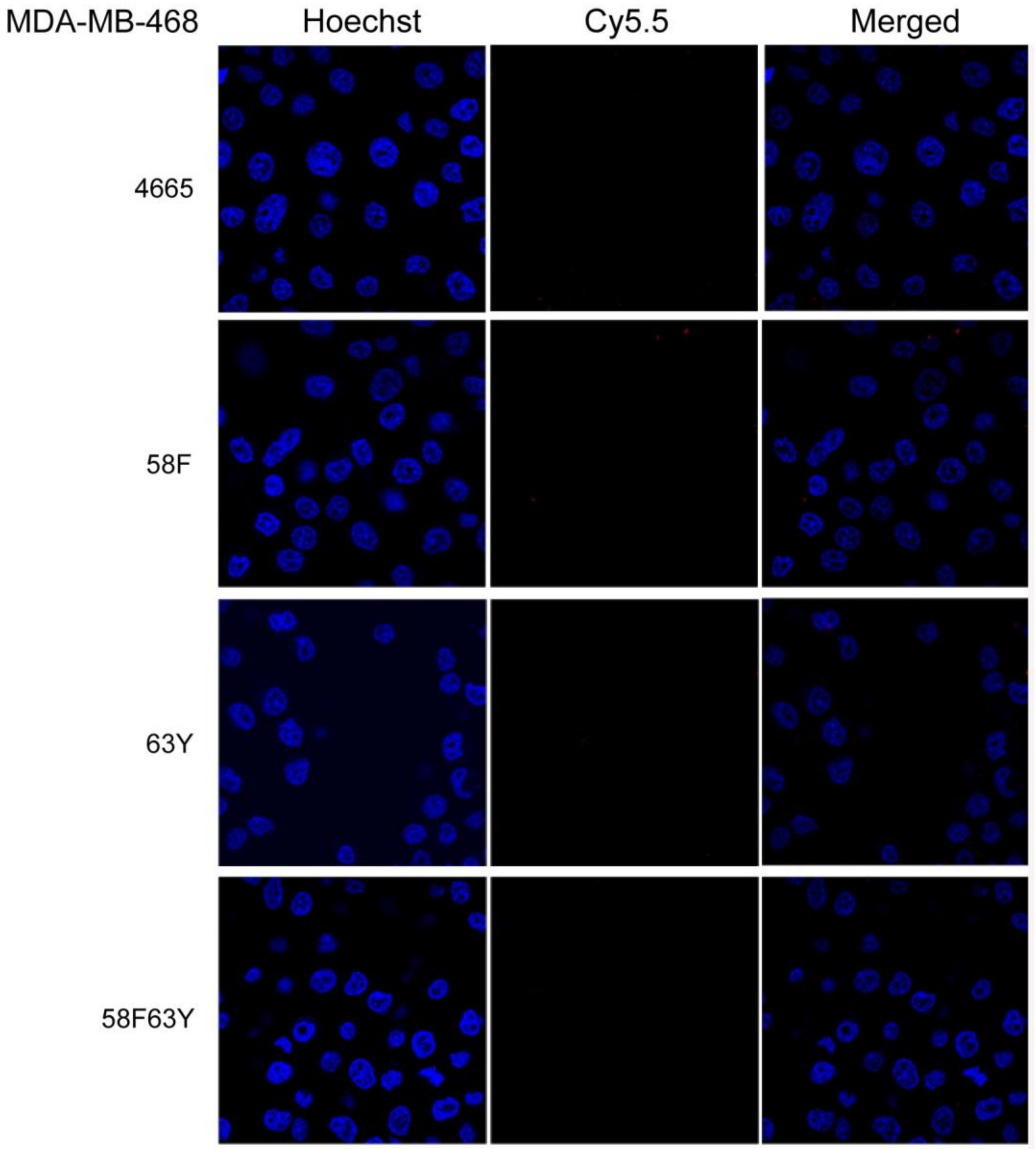

S3 Fig. Confocal fluorescence imaging analysis of the MDA-MB-468 cell line with low HER2 expression after incubating with peptides labeled with Cy5.5.
