# Supplementary figures and images for: Peptide probes derived from pertuzumab by molecular dynamics modeling for HER2 positive tumor imaging
Source: PLoS Comput Biol. 2017 Apr 13;13(4):e1005441. doi: 10.1371/journal.pcbi.1005441 (PMC5390981; doi:10.1371/journal.pcbi.1005441)

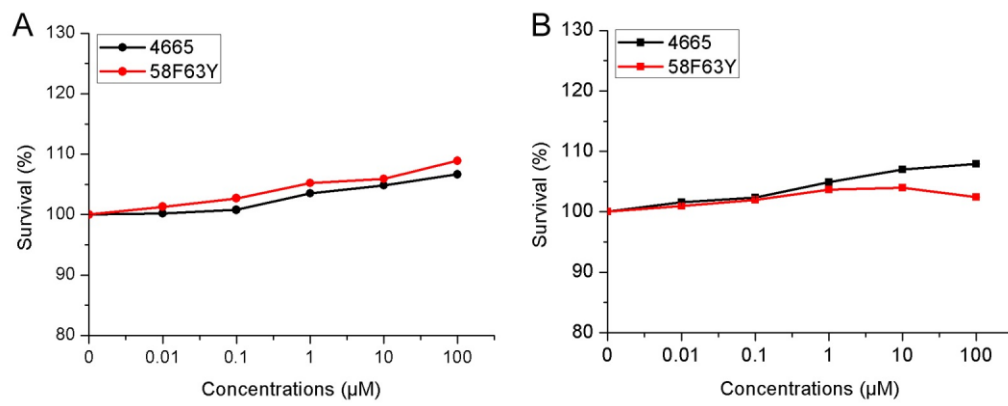

**S4 Fig. Toxicity of the peptides to HUVEC (A) and SKBR3 (B).** 4665 and 58F63Y shows no toxicity to both cell lines.

Supplement: S4 Fig — Toxicity of the peptides to HUVEC (A) and SKBR3 (B). 4665 and 58F63Y shows no toxicity to both cell lines. (PDF) [file pcbi.1005441.s004.pdf]
