# Supplementary material for: Peptide probes derived from pertuzumab by molecular dynamics modeling for HER2 positive tumor imaging
Source: PLoS Comput Biol. 2017 Apr 13;13(4):e1005441. doi: 10.1371/journal.pcbi.1005441 (PMC5390981; doi:10.1371/journal.pcbi.1005441)
Supplement: S5 Fig — (A) HER2/4665, (B) HER2/58F, (C) HER2/63Y, (D) HER2/55V, (E) HER2/58F63Y, and (F) HER2/55V63Y. The RMSD values become stable after about 5000ps in the MD trajectories, indicating the convergence of each peptide structure towards an equilibrium state. (PDF) [file pcbi.1005441.s005.pdf]

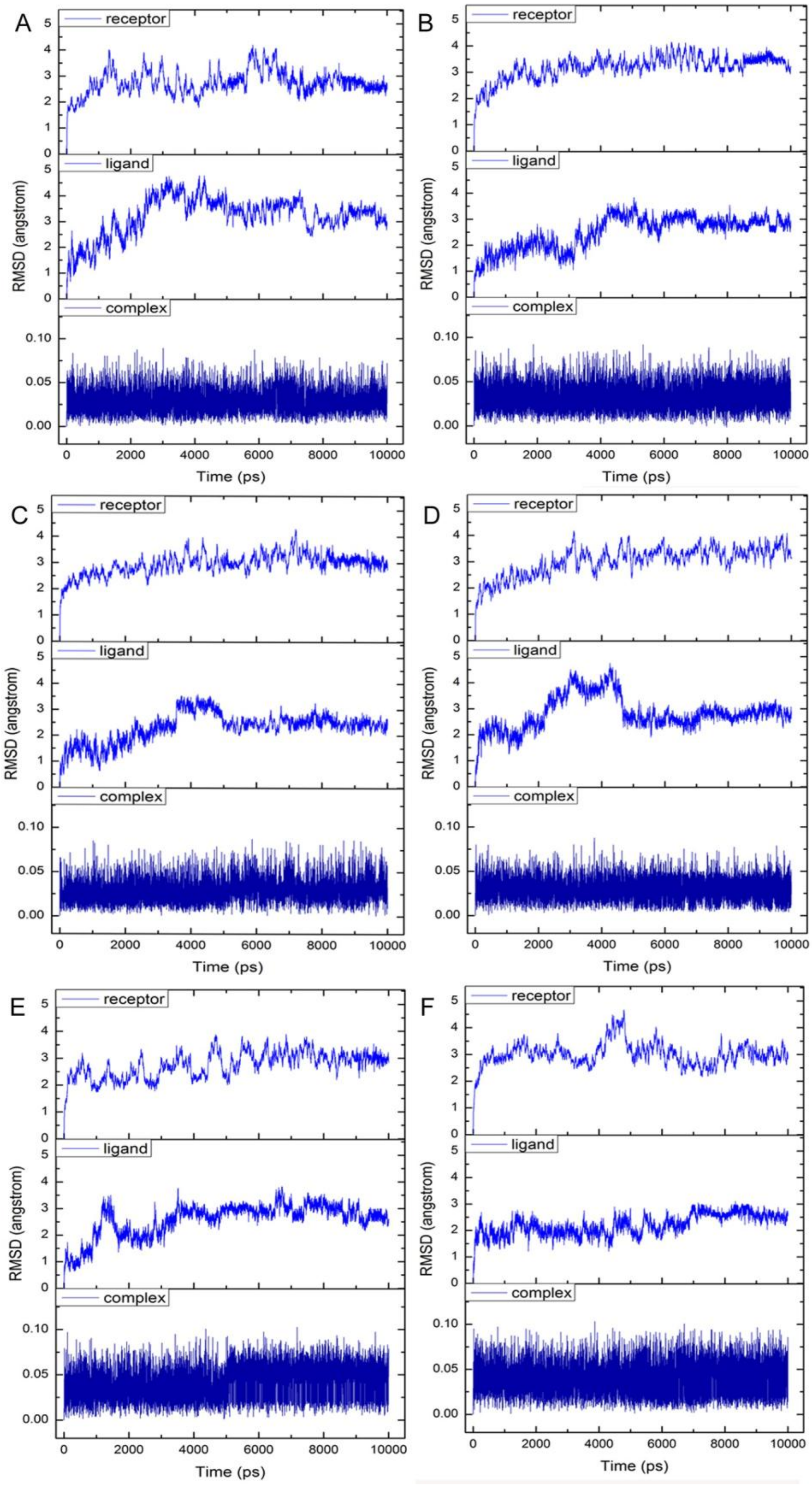

S5 Fig. Backbone RMSDs as a function of time for the initial and successive structures of HER2/peptides complexes in MD trajectories. (A) HER2/4665, (B) HER2/58F, (C) HER2/63Y, (D) HER2/55V, (E) HER2/58F63Y, and

(F) HER2/55V63Y. The RMSD values become stable after about 5000ps in the MD trajectories, indicating the convergence of each peptide structure towards an equilibrium state.
