# Supplementary material for: Peptide probes derived from pertuzumab by molecular dynamics modeling for HER2 positive tumor imaging
Source: PLoS Comput Biol. 2017 Apr 13;13(4):e1005441. doi: 10.1371/journal.pcbi.1005441 (PMC5390981; doi:10.1371/journal.pcbi.1005441)
Supplement: S6 Fig — HER2/4665 (A), HER2/58F (B), HER2/63Y (C), HER2/55V (D), HER2/58F63Y (E), and HER2/55V63Y (F). Gray cartoons are the HER2 protein, and blue cartoons are the mutant peptides. Orange spots show the main contributing residues of each peptide interacting with HER2 protein. (PDF) [file pcbi.1005441.s006.pdf]

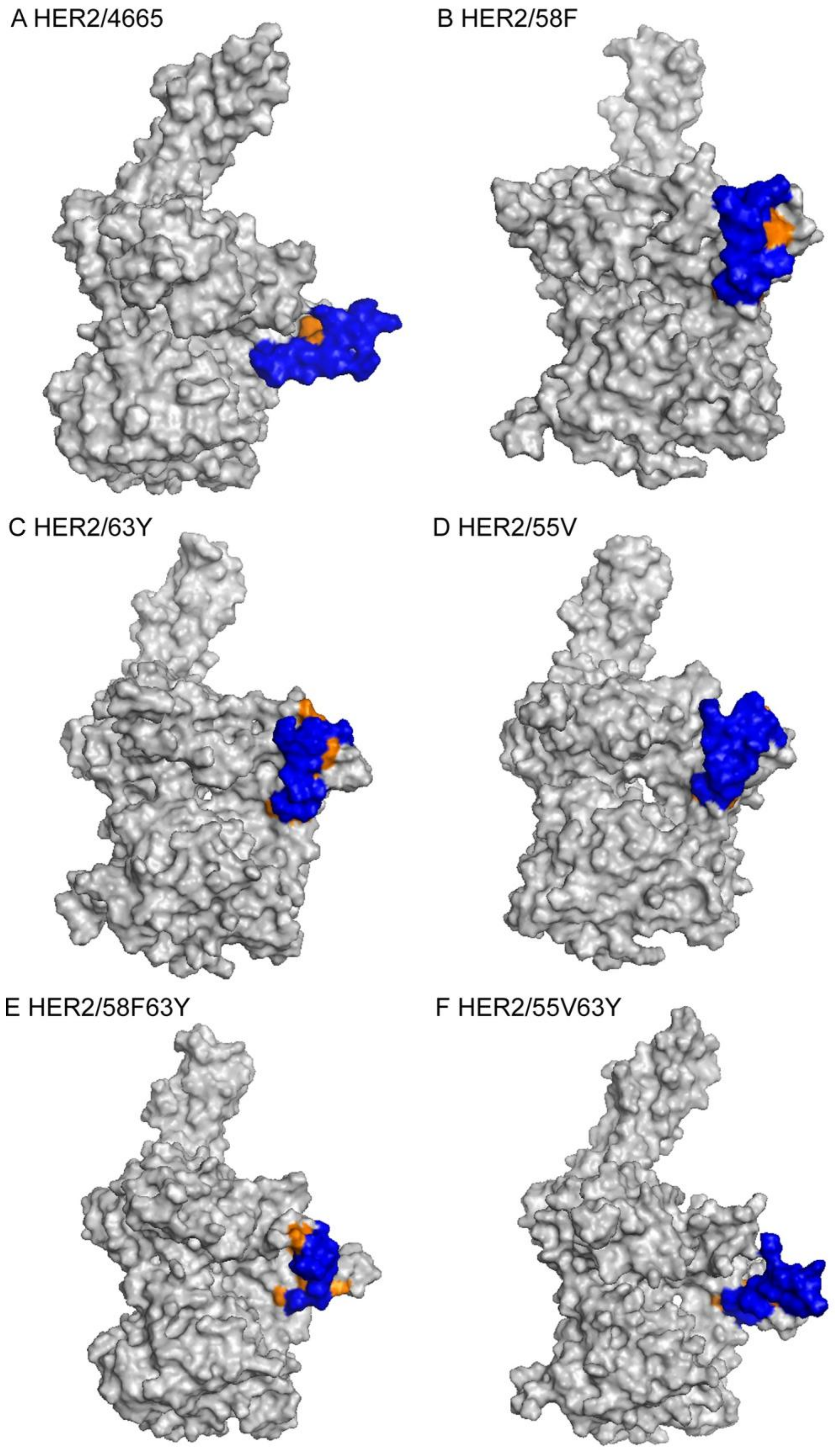

S6 Fig. Comparison of the interactions of HER2/peptides. HER2/4665 (A), HER2/58F (B), HER2/63Y (C), HER2/55V (D), HER2/58F63Y (E), and HER2/55V63Y (F). Gray cartoons are the HER2 protein, and blue cartoons are the

mutant peptides. Orange spots show the main contributing residues of each peptide interacting with HER2 protein.
