# Supplementary material for: Peptide probes derived from pertuzumab by molecular dynamics modeling for HER2 positive tumor imaging
Source: PLoS Comput Biol. 2017 Apr 13;13(4):e1005441. doi: 10.1371/journal.pcbi.1005441 (PMC5390981; doi:10.1371/journal.pcbi.1005441)
Supplement: S7 Fig — HER2/4665 (A), HER2/58F (B), HER2/63Y (C), HER2/55V (D), HER2/58F63Y (E), and HER2/55V63Y (F). Green bars in the figure represent binding free energy <–3 kcal/mol. Therefore, each mutant shown in B to F has more than three residues that contribute to the HER2/peptide complexes. (PDF) [file pcbi.1005441.s007.pdf]

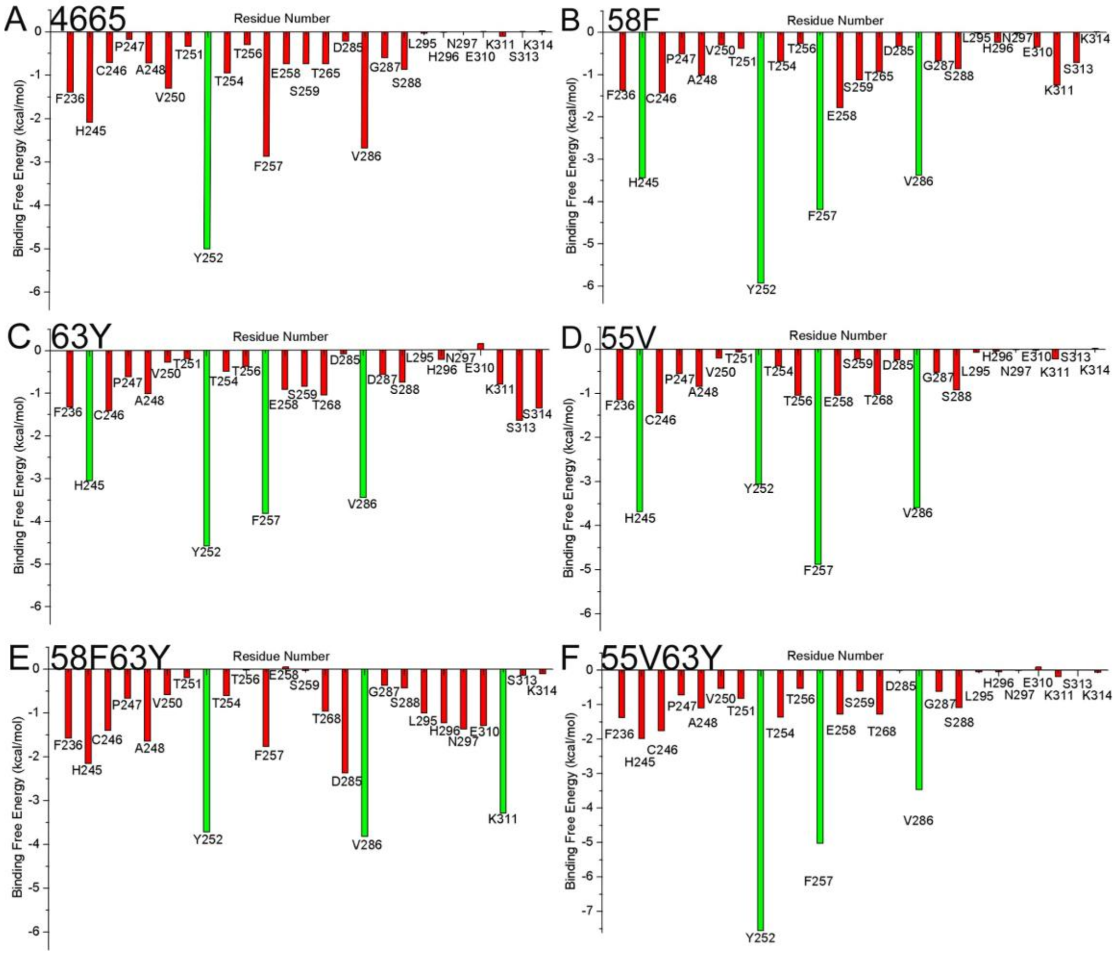

S7 Fig. Binding free energy decomposition for key residues of the HER2 protein in the six complexes. HER2/4665 (A), HER2/58F (B), HER2/63Y (C), HER2/55V (D), HER2/58F63Y (E), and HER2/55V63Y (F). Green bars in the figure represent binding free energy <–3 kcal/mol. Therefore, each mutant shown in B to F has more than three residues that contribute to the HER2/peptide complexes.
