# Supplementary material for: Peptide probes derived from pertuzumab by molecular dynamics modeling for HER2 positive tumor imaging
Source: PLoS Comput Biol. 2017 Apr 13;13(4):e1005441. doi: 10.1371/journal.pcbi.1005441 (PMC5390981; doi:10.1371/journal.pcbi.1005441)
Supplement: S8 Fig — 4665/HER2 (A), 58F/HER2 (B), 63Y4665/HER2 (C), 55V/HER2 (D), 58F63Y/HER2 (E), and 55V63Y/HER2 (F). Blue cartoons stand for mutant peptides. Key residues (binding free energy <–1 kcal/mol) in HER2 protein are shown as yellow sticks. (PDF) [file pcbi.1005441.s008.pdf]

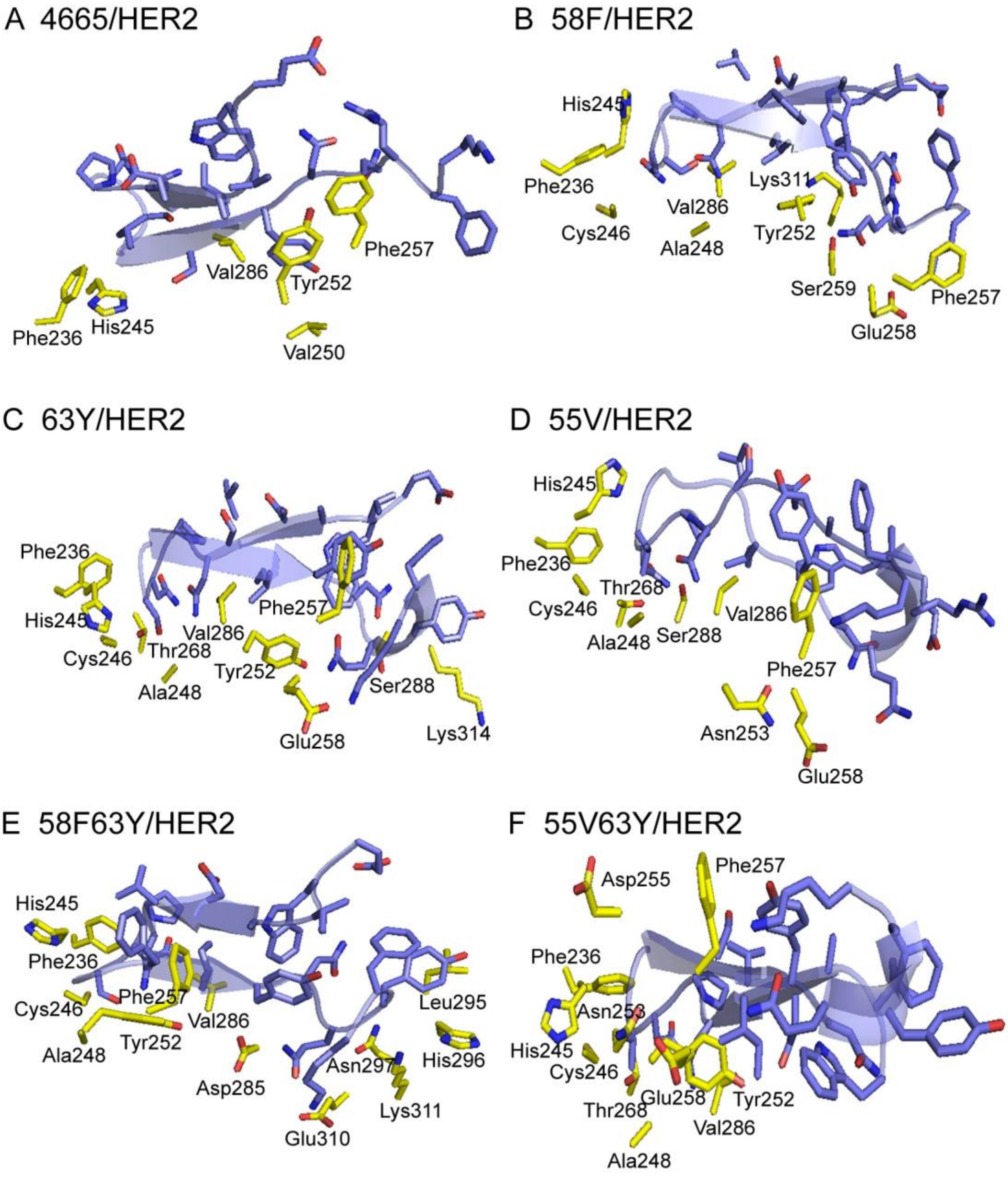

S8 Fig. Binding models of HER2/peptides complexes: 4665/HER2 (A), 58F/HER2 (B), 63Y4665/HER2 (C), 55V/HER2 (D), 58F63Y/HER2 (E), and 55V63Y/HER2 (F). Blue cartoons stand for mutant peptides. Key residues (binding free energy <–1 kcal/mol) in HER2 protein are shown as yellow sticks.
