# Supplementary material for: Peptide probes derived from pertuzumab by molecular dynamics modeling for HER2 positive tumor imaging
Source: PLoS Comput Biol. 2017 Apr 13;13(4):e1005441. doi: 10.1371/journal.pcbi.1005441 (PMC5390981; doi:10.1371/journal.pcbi.1005441)
Supplement: S1 Table — (PDF) [file pcbi.1005441.s009.pdf]

S1 Table. Numbers of water molecules and Na+ ions added in all simulation systems.

	Water molecule	Na^+
**HER2/4665**	37422	13
**HER2/48F**	37418	13
**HER2/48M**	37419	13
**HER2/48W**	37420	13
**HER2/48Y**	37420	13
**HER2/49M**	37422	13
**HER2/49Y**	37422	13
**HER2/50A**	37424	12
**HER2/50E**	37418	13
**HER2/50G**	37426	12
**HER2/50K**	37419	11
**HER2/50N**	37423	12
**HER2/50Q**	37419	12
**HER2/50R**	37418	11
**HER2/51H**	37423	13
**HER2/51Y**	37423	13
**HER2/55A**	37422	13
**HER2/55F**	37417	13
**HER2/55M**	37422	13
**HER2/55P**	37422	13
**HER2/55V**	37422	13
**HER2/55W**	37416	13
**HER2/55Y**	37417	13
**HER2/56F**	37417	13
**HER2/56M**	37420	13
**HER2/56V**	37421	13
**HER2/56Y**	37416	13
**HER2/57A**	37422	13
**HER2/57L**	37421	13
**HER2/57M**	37422	13
**HER2/57V**	37422	13
**HER2/58F**	37420	13
**HER2/58H**	37421	13
**HER2/58M**	37420	13
**HER2/58W**	37420	13
**HER2/58Y**	37420	13

**HER2/59K**	37420	12
**HER2/59L**	37422	13
**HER2/59M**	37419	13
**HER2/59R**	37418	12
**HER2/59Y**	37419	13
**HER2/60W**	37420	13
**HER2/61Q**	37422	13
**HER2/62F**	37421	13
**HER2/62M**	37421	13
**HER2/62W**	37422	13
**HER2/62Y**	37421	13
**HER2/63F**	36438	14
**HER2/63M**	36442	14
**HER2/63N**	36443	14
**HER2/63Q**	36442	14
**HER2/63V**	36443	14
**HER2/63W**	36434	14
**HER2/63A**	36422	14
**HER2/63Y**	36494	14
**HER2/64K**	37425	12
**HER2/64N**	337425	13
**HER2/64Q**	37425	13
**HER2/64R**	37425	12
**HER2/58F63Y**	36493	14
**HER2/55M56M**	37420	13
**HER2/55M56Y**	37416	13
**HER2/55M57A**	37422	13
**HER2/55M57V**	37422	13
**HER2/55M58F**	37420	13
**HER2/55M63V**	36443	14
**HER2/55M63W**	36434	14
**HER2/55M63Y**	36494	14
**HER2/55V56M**	37420	13
**HER2/55V56Y**	37416	13
**HER2/55V57A**	37422	13
**HER2/55V57V**	37422	13
**HER2/55V58F**	37420	13
**HER2/55V58H**	37421	13
**HER2/55V60W**	37420	13
**HER2/55V63V**	36443	14

**HER2/55V63W**	36434	14
**HER2/55V63Y**	36494	14
**HER2/56M57A**	37420	13
**HER2/56M57V**	37420	13
**HER2/56M58F**	37418	13
**HER2/56M58H**	37419	13
**HER2/56M60W**	37418	13
**HER2/56M63V**	36440	14
**HER2/56M63W**	36431	14
**HER2/56M63Y**	36490	14
**HER2/56Y57A**	37416	13
**HER2/56Y57V**	37416	13
**HER2/56Y58F**	37414	13
**HER2/56Y58H**	37415	13
**HER2/56Y60W**	37414	13
**HER2/56Y63V**	36438	14
**HER2/56Y63W**	36429	14
**HER2/57A58F**	37420	13
**HER2/57A58H**	37421	13
**HER2/57A60W**	37420	13
**HER2/57A63V**	36442	14
**HER2/57A63W**	36433	14
**HER2/57A63Y**	36493	14
**HER2/57V58F**	37420	13
**HER2/57V58H**	37421	13
**HER2/57V60W**	37420	13
**HER2/57V63V**	36442	14
**HER2/57V63W**	36433	14
**HER2/57V63Y**	36493	14
**HER2/58F60W**	37418	13
**HER2/58F63V**	36442	14
**HER2/58F63W**	36433	14
**HER2/58H60W**	37419	13
**HER2/58H63V**	36444	14
**HER2/58H63W**	36435	14
**HER2/58H63Y**	36495	14
**HER2/60W63V**	36439	14
**HER2/60W63W**	36430	14
**HER2/60W63Y**	36491	14
